# In vitro bacteriological effect of tri-beveled needle electrolysis against *Staphylococcus aureus*

**DOI:** 10.1038/s41598-022-15666-w

**Published:** 2022-07-06

**Authors:** José Antonio García-Vidal, Jesús Salinas, Nieves Ortega, Pilar Escolar-Reina, Fabio Camacho-Alonso, Francesc Medina-Mirapeix

**Affiliations:** 1 Department of Physiotherapy, University of Murcia, Campus de Espinardo, 30100 Murcia, Spain; 2 Research Group Fisioterapia y Discapacidad, Institute of Biomedical Research (IMIB)-Virgen de la Arrixaca University Clinical Hospital, 30120 El Palmar, Murcia, Spain; 3 Department of Animal Health, Faculty of Veterinary, University of Murcia, Campus de Espinardo, 30100 Murcia, Spain; 4 Department of General Dentistry and Implants, Faculty of Medicine and Dentistry, University of Murcia, 30008 Murcia, Spain

**Keywords:** Microbiology techniques, Translational research, Infection, Inflammation

## Abstract

Percutaneous needle electrolysis using tri-beveled needles with a specific protocol (5 mA applied for 25 s) has demonstrated to provoke a clinical reduction of recurrent bacterial infections in mammary fistulas. However, the bactericidal effect of needle electrolysis in this pathology remains theoretical. This in vitro study evaluated the bactericidal effect of this protocol and whether it changed when introducing small variations. *Staphylococcus aureus* were generated in saline solution (9 Log_10_ CFU/mL) and treated in three different experiments including the main protocol and introducing variations in needle gauge, intensity, and total dosage, respectively. After 24 h, the viable cell count showed that the protocol had an average reduction of 5 log_10_ CFU/ml compared to the control group. While variations in needle gauge did not modify this effect, variations in current intensity or dosage did. This study demonstrated that the bacterial effect was greater by increasing either current intensity or total dosage, and it decreased with substantial reductions of these parameters.

## Introduction

The application of a galvanic current produces a physical phenomenon known as electrolysis which has a recognized power of disinfection against viruses and bacteria^1^. Through different types of electrodes, electrolysis has been used for disinfection of hands and surfaces^2^, medical and dental supplies^3,4^, surgical procedures^5^, food^6,7^ and even industrially, in swimming pools and wastewater^8,9^.

Traditionally, physiotherapists have also used the application of a galvanic current for other additional therapeutic purposes by using either electrodes or acupuncture needles. Electrodes have been used for analgesic purposes^10^ and for the local use of drugs by iontophoresis^11^. Acupuncture needles have mainly been applied to generate local inflammation, tissue regeneration and analgesia, for the treatment of musculoskeletal pathologies associated with degenerative processes, such as chronic tendinopathies^12,13^. This application of galvanic current by needles has been called needle electrolysis (NE), which is considered to be a minimally invasive approach that generates different alkaline molecules, capable of generating a non-thermal electrochemical ablation by cathodic flow directly into the affected tissue^12^. Recently, a bactericidal effect has been demonstrated in vitro with this type of needle used in acupuncture^14^.

It has been speculated that the application of electrolysis through percutaneous tri-beveled needles could also have a bactericidal effect in pathologies associated with recurrent bacterial infections related to the skin such as mammary fistulas^15,16^, in which *Staphylococcus aureus* is the most common germ involved^17^. Nonetheless, until now, the bactericidal effect of NE using tri-beveled needles in this type of pathology remains theoretical^16^. Since Berna-Serna et al.^16^ demonstrated the clinical effectiveness of their protocol for the treatment of mammary fistula, a current intensity of 5 mA applied for 25 s distributed in five pulses of five seconds each through a 14G tri-beveled needle, representing an electric charge dose of 0.125 Coulombs (C), we hypothesized that both this protocol of treatment as well as proxy variations of parameters could have a bactericidal effect.

The aims of this in vitro study were, firstly, to determine whether the protocol reported by Berná-Serna et al.^16^ for mammary fistula has a bactericidal effect against *S. aureus*. Secondly, to evaluate whether variations in needle gauge and dosage over a specific protocol are able to increase or reduce the bactericidal effect of NE. The specific research questions we addressed for this first aim were: (a) Is there a difference in the bactericidal effect for different needle gauges using the same dosage?; (b) Is there a difference in the bactericidal effect for different current intensities applied in equivalent doses of electric charge (0.125 C)?; (c) Is there a difference in the bactericidal effect when different doses of electric charge are applied?.

## Materials and methods

### Bacterial preparation

**Figure 1:**
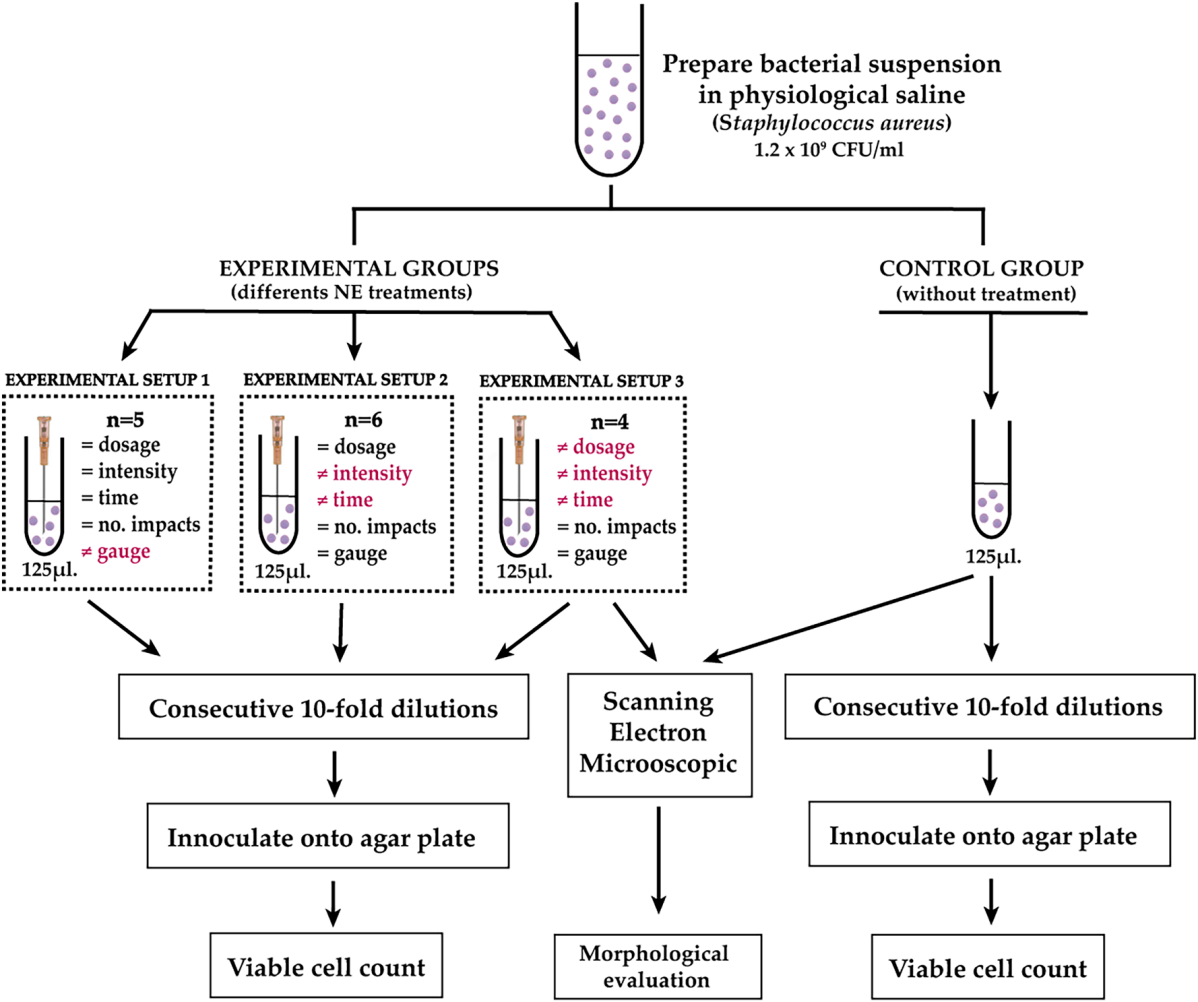
Flow diagram showing an experimental design to determine the bactericidal effect of NE used in the different in vitro experiments.

The *Staphylococcus aureus* strain ATCC 25923, purchased from the Spanish Type Culture Collection, was used in the present study. The microorganism was cultured in Petri dishes (90 × 15 mm) containing Mueller–Hinton medium (bioMérieux, Spain), and incubated for 24 h at 37 °C. For each experimental setup, several colonies (8 to 12 depending on size) were collected and transferred onto 5 mL of sterile physiological saline solution (0.9% NaCl in water, Sigma-Aldrich, Spain). To standardize the number of bacteria in the suspension, we used a McFarland suspension of 4, which equals approximately 1.2 × 10^9^ Colony-Forming Units (CFU) per milliliter (or OD = 1 at 540 nm). The volume of bacterial suspension added to tubes is mentioned in Fig. 1 (125 ml).

### Electrical current application

**Figure 2:**
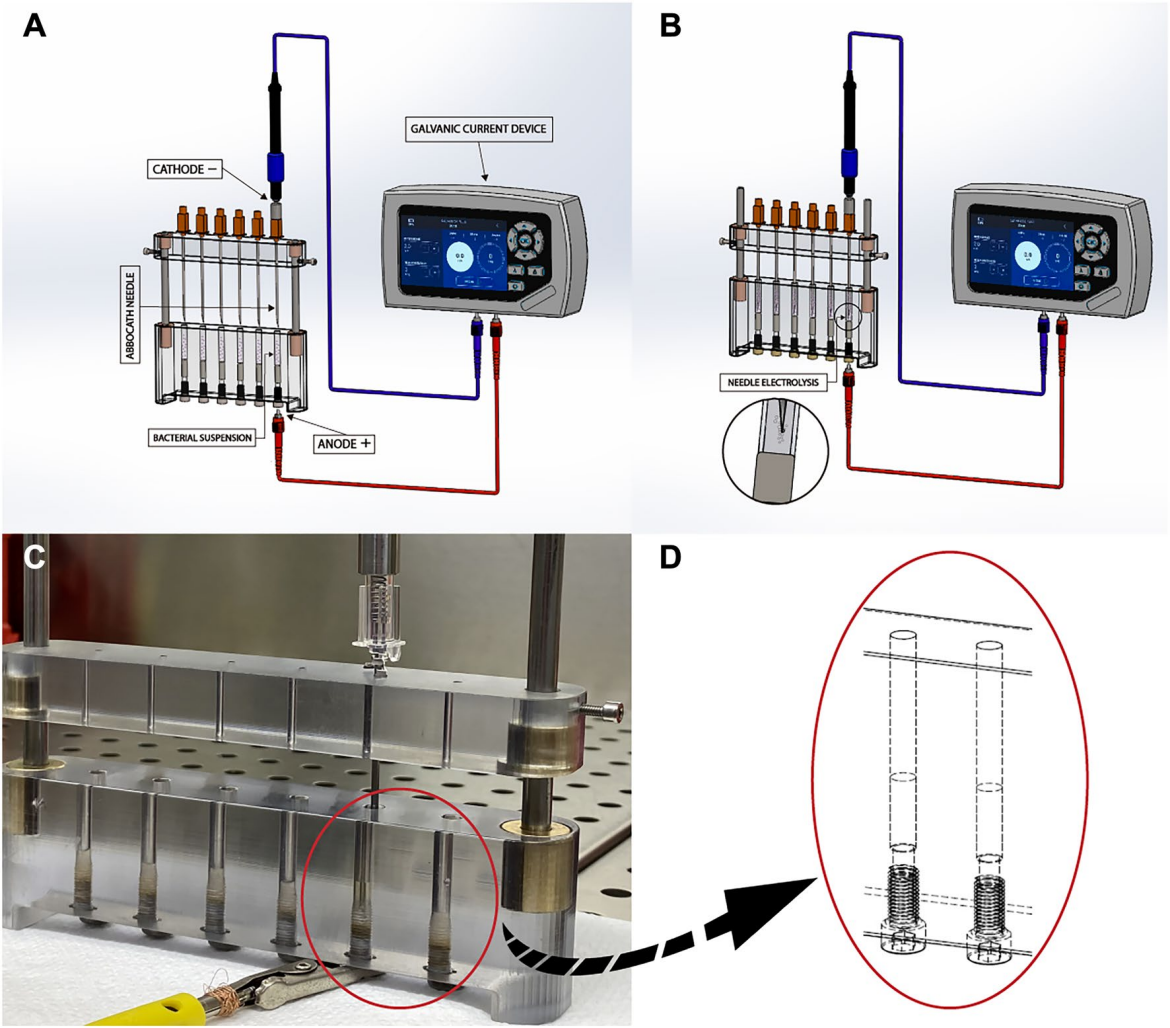
(**A**) Preparing the elements for conducting the experiments**.** (**B**) NE application through a tri-beveled needle on a bacterial suspension of *S. aureus*. (**C**) In vitro device for application of needle electrolysis designed to simulate in vivo application conditions. (**D**) Schematic zoom of the receptacle where the cathodic flow of the electrolysis to the bacteria is generated.

For the galvanic current generator, we used a Galvani-K® device (Medi-K New Solutions, Murcia, Spain), which produces a continuous galvanic current (Fig. 2A,B) through a tri-beveled needle (Braun© Introcan Safety 14G 2.2 mm × 50 mm) as a cathode, whereas the anode was in contact with the bacterial suspension through a conductive metal link. A special device was designed and patented (ES2793098) for the in-vitro application of needle electrolysis (Fig. 2C). Figure 2D shows the receptacle of this device used to perform needle electrolysis on bacteria.

### Experimental designs

To analyze the effect of NE on *S. aureus*, three different experiments were carried out (Fig. 1):

- Experimental setup 1: To verify whether the needle gauge (G) has any influence on the bactericidal effect of the electrical galvanic currents, five tri-beveled needle thicknesses were used (14 G, 16 G, 18 G, 20 G and 24 G, with 24 G being the thinnest needle and 14 G the thickest) maintaining the total current dose (0.125 C). In all cases, five pulses of 5 mA were applied for 5 s each.
- Experimental setup 2: To verify whether the bactericidal effect depends on the intensity of the galvanic current or the time of application, we kept a constant electric current dose of 0.125 C. For this purpose, a 14 G tri-beveled needle was used, subjecting the bacterial suspensions to five consecutive impacts with the following treatments: 0.4 mA for 62.5 s, 1 mA for 25 s, 2 mA for 12.5 s, 3 mA for 8.3 s, 4 mA for 6.2 s and 5 mA for 5 s.
- Experimental setup 3: To verify whether there is a relationship between bactericidal effects and the total dose of electric charge, two different intensities (3 and 5 mA) and times (5 and 10 s) were selected, always applying five consecutive impacts to the bacterial suspensions using a 14 G tri-beveled needle. This represents galvanic current doses of 0.075, 0.125, 0.15 and 0.25 C, depending on the combination of intensity and time applied.

The specific dosage with a 14G needle used in the protocol by Berná-Serna et al. (2020) for mammary fistula was tested in each experimental setup. All experiments (including control groups without treatment) were performed at least three times to verify the reproducibility of the results.

### Bacteriological evaluation

Following all experiments, decimal serial dilutions (from 10^–1^ to 10^–7^) were prepared. Subsequently, 100 µL of each dilution were seeded on the surface of Mueller–Hinton dishes (bioMérieux) in triplicate and incubated at 37 °C for 24 h (Fig. 1). The number of colonies was counted at the appropriate dilution and the number of CFU/mL were calculated and transformed in log_10_ scale.

### Scanning electron microscopic evaluation

This procedure was performed exclusively for the groups of experimental setup 3 that received the lowest and highest dose of galvanic current. To analyze the morphological changes, three biofilm samples (control and treated with 0.075 or 0.25 C) were processed for scanning electron microscopic evaluation. The samples were washed twice with phosphate-buffered saline (pH = 7.2), fixed with 2.5% glutaraldehyde overnight, dehydrated by immersion in acetonitrile solutions of increasing concentrations (50%, 70%, 80%, 90%, and 100% twice for 20 min each), dried using the critical drying point with the Leica EM CPD030 (Leica Microsystems, Heidelberg GmbH, Mannheim, Germany), sputter coated with platinum using the Leica EM ACE600 (Leica Microsystems, Heidelberg GmbH, Mannheim, Germany), and observed with a scanning electron microscope JEOL JSM-6100 (JEOL, Tokyo, Japan) at 20 kV. The morphology of the bacterial cells was described using the morphological description reported by Cheng et al. (2016): normal morphology (round cells with bright surface and without any apparent cell lysis), or abnormal morphology (flattened and shrunken cells with rough surface, and lysed cells).

### Statistical analysis

A one-way between-groups analysis of variance was conducted to explore the impact of needle gauge on bacterial death levels (experiment number 1), and again to explore the impact of variations of current intensity keeping a same dose of electric charge (number 2), and finally the impact of different dose of electric charge (number 3). All experiments were performed at least three times. We also employed post-hoc comparisons with the Tukey HSD. Additionally, in experiment number 3, one two-way between-groups analysis of variance was conducted to explore the possibility of interaction effect between current intensity and time. Differences were considered significant at *p* < 0.05. Statistical analysis of all data was performed using SPSS for Windows (version 24, SPSS Inc., Chicago, IL). The data were expressed as the mean with the confidence interval (CI).

## Results

### Relevance of the tri-beveled needle gauge on the bactericidal effect of the galvanic currents

**Figure 3:**
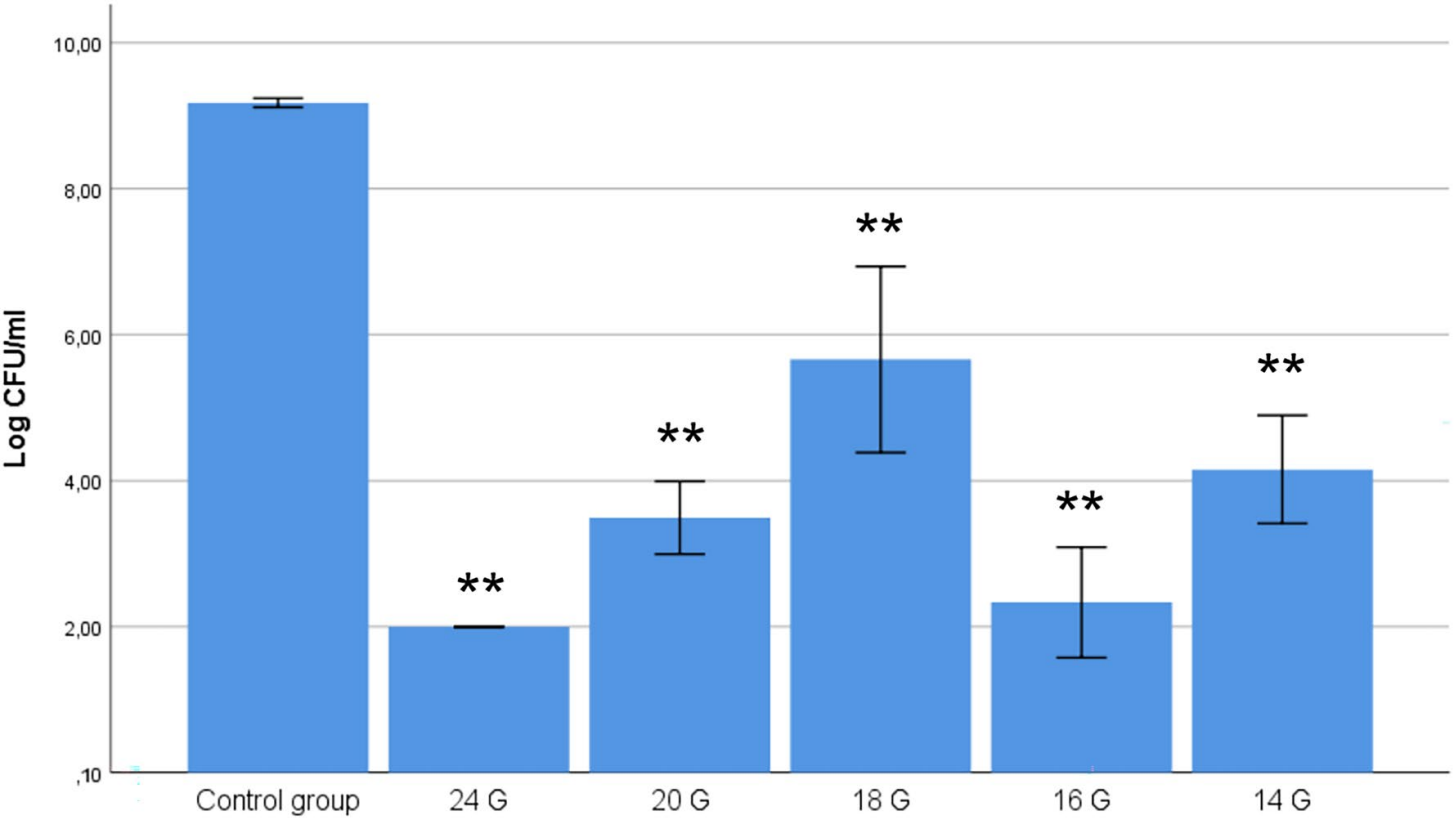
The relevance of needle gauge has an influence on the bactericidal effect of the galvanic currents on *S. aureus*. Data were expressed as mean with CI of the number of viable log_10_ CFU/ml. (**: statistical significance *p* < 0.001 in relation to the control group).

In experimental setup 1, the influence of the gauge of the needles on the bactericidal effect of the galvanic currents was analyzed (Fig. 3). There was a statistically significant difference at the *p* < 0.05 level in the concentration of these six groups: F(5,12) = 261.9, *p* < 0.001. Post-hoc comparisons using the Tukey HSD test indicated that the mean of all experimental groups differed significantly in relation to the control group. The lowest bacterial concentration achieved was 2.0 log_10_ CFU/ml (in 24G), which represented a reduction of 7 log_10_ CFU/ml. Whereas among some groups there were no statistically significant differences (24 G and 16 G (*p* = 0.707), and 20 G and 14 G (*p* = 0.117), the remaining groups did show differences. Nevertheless, these associations were not gauge dependent.

### Relevance of the intensity and time of application of galvanic currents on the bactericidal effect

**Figure 4:**
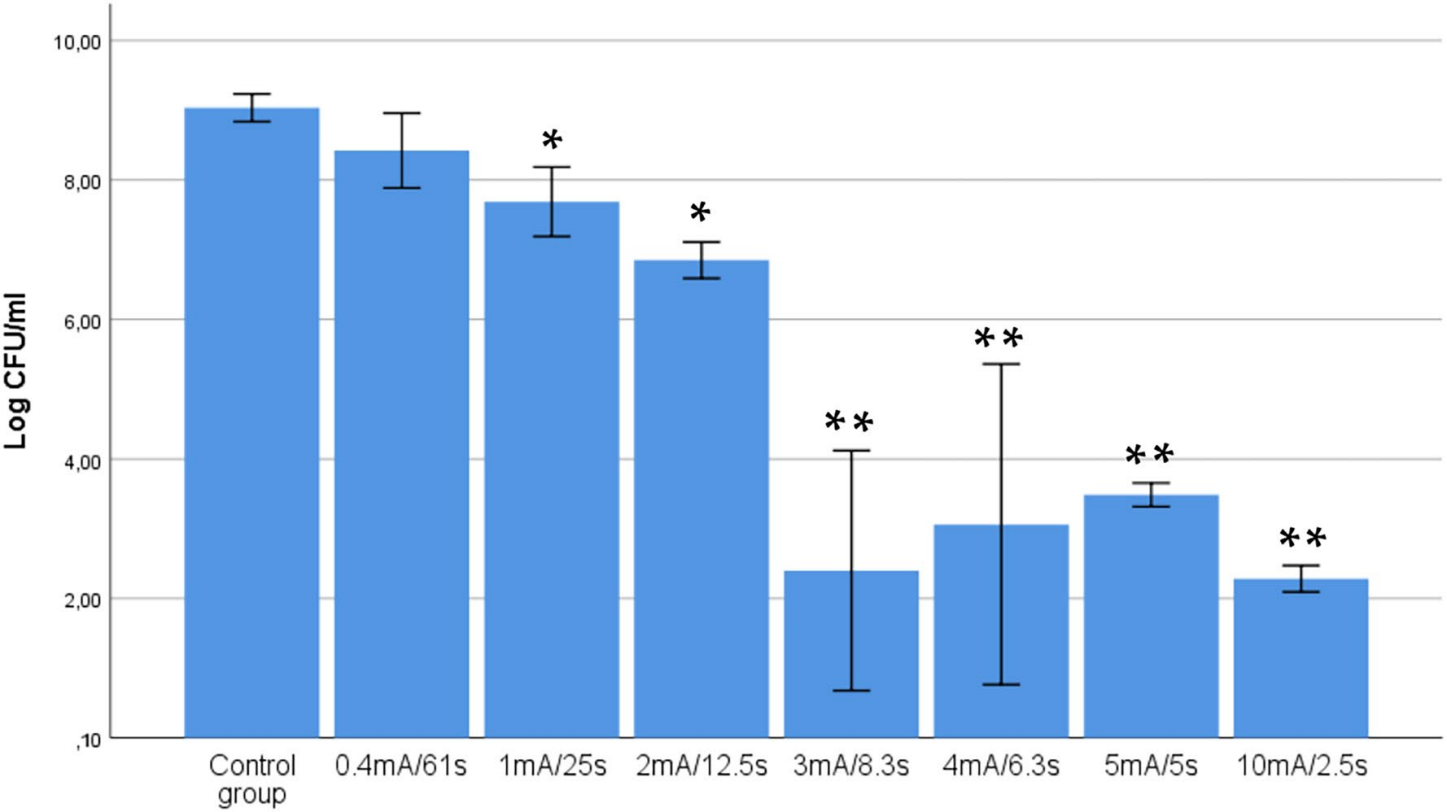
Relevance of the intensity and time of application of a constant dose of 0.125 C of galvanic currents on the bactericidal effect on *S. aureus.* 14 G tri-beveled needle (2.2 mm × 50 mm) was used, subjecting the bacterial suspensions to five consecutive impacts with the following treatments: 0.4 mA for 62.5 s, 1 mA for 25 s, 2 mA for 12.5 s, 3 mA for 8.3 s, 4 mA for 6.2 s and 5 mA for 5 s. Data were expressed as mean with CI of the number of viable log_10_ CFU/ml. (*: statistical significance *p* < 0.05 in relation to the control group; **: statistical significance *p* < 0.001 in relation to the control group).

In experimental setup 2, the viable bacterial concentrations of the control group and the seven experimental groups received equivalent doses of 0.125 C, by applying different current intensities during different times. In both this experiment and in the remaining experiments, we used 14G because this was the gauge used in previous clinical trials^15,16^ and because experimental setup 1 showed a non-dependent association between gauge and bacteriological effect. The results were compared (Fig. 4) and statistically significant differences at *p* < 0.05 were detected in the bacterial concentration of these eight groups: F(7,16) = 135.9, *p* < 0.001. The means in the log_10_ CFU/ml of all the groups, except the 0.4 mA group, differed statistically from the control group (*p* < 0.05).

Bacterial concentrations were substantially lower with higher intensities. The lowest bacterial concentration achieved was 2.2 log_10_ CFU/ml (3 mA and 10 mA), which represented a reduction 6.8 log_10_ CFU/ml compared to the control. The minimal current intensity that provided the highest reduction was 3 mA.

### Relevance of the dose of electric charge of galvanic currents on the bactericidal effect

**Figure 5:**
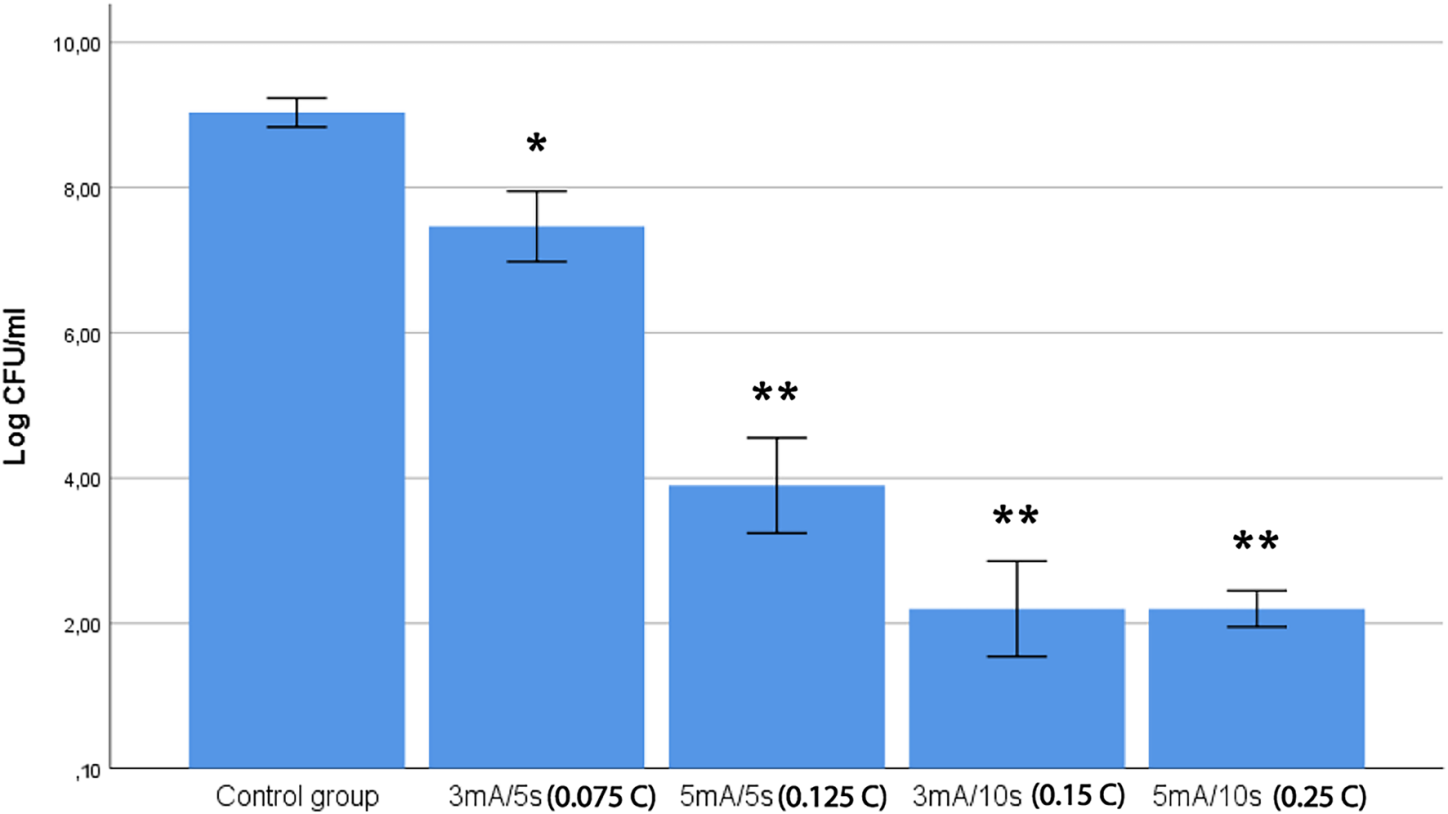
Relevance of the dose of the galvanic currents on the bactericidal effect on *S. aureus*. Data were expressed as mean with CI of the number of viable log_10_ CFU/ml. NE parameters were expressed as “intensity (mA)/ time (seconds)”. Two different intensities (3 and 5 mA) and times (5 and 10 s) were selected, always applying five consecutive impacts to the bacterial suspensions on a 14 G tri-beveled needle (2.2 mm × 50 mm). Doses were expressed in Coulombs (C). (*: statistical significance *p* < 0.05 in relation to the control group; **: statistical significance *p* < 0.001 in relation to control group).

In experimental setup 3, the viable bacterial concentrations of the four experimental doses of electric charge and the control group were evaluated (Fig. 5). An ANOVA test revealed a statistically significant difference in bacterial concentrations across the four dose groups F(3,10) = 755.5, *p* < 0.001. Post-hoc comparisons indicated that all groups, except these with a higher dose (0.15 C and 0.25 C), had significant differences between them.

Differences between groups with 0.075 C and 0.15 C (both with 3 mA) were statistically higher than differences than groups with 0.125 C and 0.25 C (both with 5 mA). These findings revealed a significant interaction effect between two of the times used (5 and 10 s) and two experimental intensities (3 and 5 mA) on bacterial death levels (F(1,6) = 307, *p* < 0.001). The bactericidal effect increased much more for the 3 mA than for the 5 mA when longer times were used, therefore the increased dose of electric charge produced with increasing time does not produce the same bactericidal effect at the two intensity levels analyzed.

**Figure 6:**
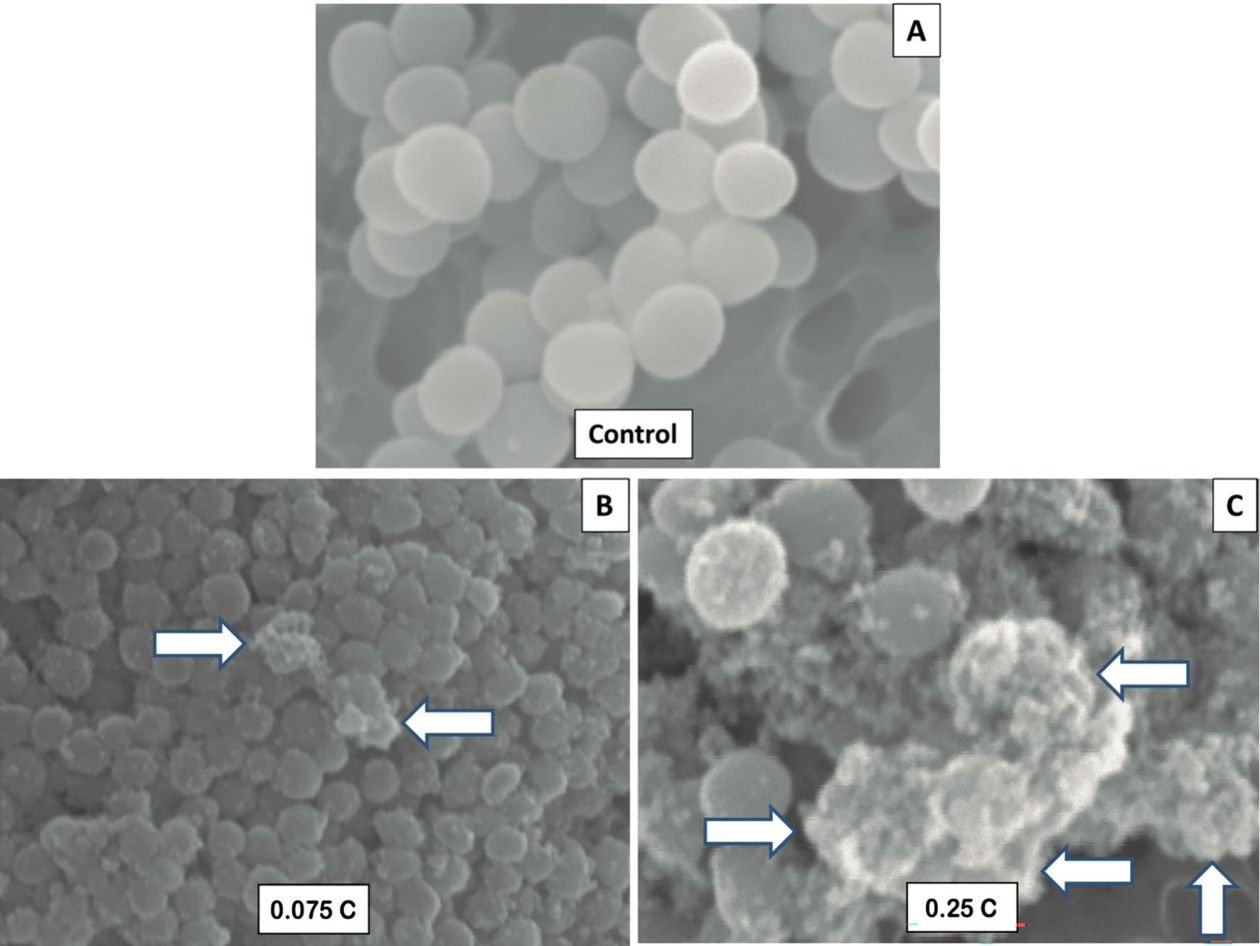
Bacterial cells morphology observed with scanning electron microscopic evaluation. (**A)** Control group with normal morphology (round cells with bright surface and without any apparent cell lysis) (10,000×). (**B)** Biofilm sample treated with 0.075 C, with abnormal morphology (flattened and shrunken cells with rough surface and with some lysed cells marked with white arrows) (8000×). (**C)** Biofilm sample treated with 0.25 C, with more abnormal bacterial cell morphology (flattened and shrunken cells with rough surface and more lysed cells marked with white arrows) (9500×).

Morphological changes in the external structure of *S. aureus* were observed between the untreated control group and 0.075 C or 0.25 C in the treated groups using a scanning electron microscope (Fig. 6). The control group showed a normal morphology described as a round cell with a bright surface and without any apparent cell lysis (Fig. 6A), whereas the group treated with 0.075 C showed an abnormal morphology with flattened and shrunken cells with a rough surface and with some lysed cells (Fig. 6B), which was much more marked in the 0.25 C treated group (Fig. 6C). The results of the morphological study agree with the results of the bacteriological count, since although the control group showed an average bacterial load of 9.08 log_10_ CFU/ml, the groups treated with 0.075 C and 0.25 C showed averages of 5.04 and 3.3 log_10_ CFU/ml, respectively (Fig. 5).

## Discussion

Our results show that the dosage used by Berná-Serna et al. (2020) in the treatment of mammary fistula by applying a current intensity of 5 mA for 25 s distributed in five pulses of five seconds each through a 14G tri-beveled needle had a significant bactericidal effect, showing an average reduction of 5 log_10_ CFU/ml compared to the control group. Furthermore, variations in needle gauge did not significantly modify this bactericidal effect. Our outcomes also revealed that the bactericidal effect can be modified either without changing the total dose of electric charge (by modifying current intensity and time) or by using different doses of electric charge. Finally, we identified that when using these two approaches for changing the bactericidal effect there is a maximum effect after which higher doses or intensities do not add a greater bactericidal effect.

To our knowledge, this is the first study to demonstrate the bactericidal effect of electrolysis using the tri-beveled needle. By applying the specific dosage used by Berna-Serna et al.^16,17^, our findings support the hypothesis of this work, which found this technique to be an effective therapeutic option in pathologies that commonly become infected, such as mammary fistulas.

Based on the results obtained, the needle gauge seems not have a lineal relationship with the level of bacterial death. Our results with needles of the smallest gauge were similar to previous in vitro studies that found good results using acupuncture needles (0.30 mm × 30 mm) with current doses of 0.125 C^14^. This equivalence between studies strengthens our affirmation.

Our results using a constant electric charge dose of 0.125 C showed that a current intensity of 0.4 mA had no bactericidal effect, finding that the intensity of 3 mA significantly increased the effect compared to lower intensities (1 mA and 2 mA). Moreover, this effect did not increase further with higher intensities (5 mA and 10 mA). These findings support the fact that the response to intensity changes without modifying the total dose of electric charge may be complex and seems to be non-linear in most cases. These findings highlight that a minimum current intensity threshold is necessary for achieving a bactericidal effect and that there is an optimal intensity required after which the bactericidal effect is less pronounced.

In our opinion, whether or not an optimal current intensity truly exists for each dosage, our results may help to clarify the existing debate regarding which intensity may be more appropriate. Some authors^18–20^, based on Faraday’s law of electrolysis (Q = I × t), have defended the effectiveness of dosages with low current intensity (350 µA) and long application times, whereas others, such as Valera-Garrido et al.^12^ or, more accurately, Abat et al.^13^ have endorsed the use of higher current intensities (3 mA) with shorter application times. Perhaps our results do not add evidence to the therapeutic effectiveness of galvanic current, however, they do support the bactericidal effect.

Our study also revealed that the bactericidal effect showed a non-linear dose–response relationship. This relationship increased for doses less than 0.15 C, however, greater doses did not increase the bactericidal effect (e.g., with 0.25 C). Similarly, the structural damage produced by galvanic currents on *S. aureus* clearly increased with the increase of the applied dose, which was much more severe when 0.25 C was administered. The results of the morphological study were correlated with the count of viable bacteria, since the greater the structural damage, the lower the bacterial count, which shows a greater bactericidal effect.

Our experimental setup number 3 did not determine the minimal dose of electrical charge to achieve the greatest bactericidal effect, we only determined the most likely interval (between 0.125 and 0.15 C) for a bacterial concentration of 9 log_10_ CFU/ml. Nevertheless, it is possible that other bacteria concentrations may require a different optimal dose. Considering that we also identified in a previous study that dose interacts with bacterial concentration for determining the bactericidal effect^14^, we decided to select a high concentration, which would be what we could find in a major infection. In our opinion, these two findings open up a wide range of possibilities for future studies. Determining the optimal bactericidal dose for other bacterial concentrations and with different types of bacteria could have a significant clinical impact.

The results of experimental setup 3 also showed that the effects of the current intensity appeared to interact with time of application. Thus, for example, we found that an increase of 5 s for time did not produce the same increase on the bactericidal effect at the two intensity levels analyzed (3 mA and 5 mA). Caution is therefore advised when interpreting this finding regarding its generalization to all doses until further research has examined results with lower doses. Perhaps using intensities of 3 mA, 4 mA and 5 mA could clarify these doubts.

Our findings regarding the bactericidal effect of NE are highly relevant for clinical applications of this technique. Thus, this demonstrated effect could be applied to pathologies that present an infection similar to mammary fistula, such as hidradenitis suppurativa^21^. Furthermore, since we found that the bactericidal effect does not depend on the needle gauge, we could use NE in pathologies of multiple locations and different sizes, from mammary fistulas which can have a different diameter and volume^16,17^, to hidradenitis suppurativa which are usually located in small areas of the armpits or inguinal fold^22^. In addition, since previous authors^14^ have pointed out that it can have a needle dragging effect in percutaneous applications, these findings offer greater safety to this novel technique.

A limitation of the present study was that the bactericidal effect of NE was only tested in only one strain of *S. aureus*, and therefore further studies are necessary to determine the effect of this technique on other species of microorganisms, either bacteria or yeasts, including clinical strains. In addition, in future studies, it would be interesting to evaluate the effect of NE on bacterial cultures diluted in solutions with closer chemical composition to body fluids.

In conclusion, electrolysis applied with a tri-beveled needle has a bactericidal effect against *S. aureus* and the level of this effect is dependent of the total dose of electric charge. The specific dose used by Berná-Serna et al.^16^ has that effect. Moreover, within this specific dose, there is both a minimum threshold of intensity necessary to produce a detectable bactericidal effect and a maximum threshold of intensity beyond which higher intensities do not have a greater effect against *S. aureus.*

## Supplementary Information

Supplementary Information 1.

Supplementary Information 2.

Supplementary Information 3.
